# Integrating evidence on patient preferences in healthcare policy decisions: protocol of the patient-VIP study

**DOI:** 10.1186/1748-5908-8-64

**Published:** 2013-06-10

**Authors:** Carmen D Dirksen, Cecile MA Utens, Manuela A Joore, Teus A van Barneveld, Bert Boer, Dunja HH Dreesens, Hans van Laarhoven, Cees Smit, Anne M Stiggelbout, Trudy van der Weijden

**Affiliations:** 1 Department of Clinical Epidemiology and Medical Technology Assessment, Maastricht University Medical Centre, P.O. Box 5800,6202 AZ, Maastricht, the Netherlands; 2 CAPHRI, School for Public Health and Primary Care, Maastricht University, P.O. Box 616, 6200 MD, Maastricht, the Netherlands; 3 Department of Support of Professional Quality, Dutch Association of Medical Specialists, P.O. Box 20057, 3502 LB, Utrecht, the Netherlands; 4 Department of Insurance and Benefit Package, Health Care Insurance Board, P.O. Box 320, 1110 AH, Diemen, the Netherlands; 5 Department of Quality, Health Care Insurance Board, P.O. Box 320, 1110 AH, Diemen, the Netherlands; 6 De Hart&Vaatgroep, P.O. Box 300, 2501 CH, the Hague, the Netherlands; 7 Patient expert, policy advisor Dutch Genetic Alliance (VSOP), Koninginnelaan 23, Soest, 3762 DA, the Netherlands; 8 Department of Medical Decision Making, Leiden University Medical Centre, P.O. Box 9600, 2300 RC, Leiden, the Netherlands; 9 Department of General Practice, CAPHRI, School for Public Health and Primary Care, Maastricht University, P.O. Box 616, 6200 MD, Maastricht, the Netherlands

**Keywords:** Patient preference, Coverage decisions, Clinical practice guidelines, Decision framework, Taxonomy

## Abstract

**Background:**

Despite a strong movement towards active patient involvement in healthcare policy decisions, systematic and explicit consideration of evidence of this research on patient preferences seems limited. Furthermore, little is known about the opinions of several stakeholders towards consideration of research evidence on patient preferences in healthcare policy decisions. This paper describes the protocol for an explorative study on the integration of research on patient preferences in healthcare policy decisions. The study questions: to what extent research evidence on patient preferences is considered in current procedures for healthcare policy decisions; opinions of stakeholders regarding the integration of this type of evidence in healthcare policy decisions; and what could be a decision framework for the integration of such research evidence in healthcare policy decisions.

**Methods/design:**

The study is divided in three sub-studies, predominantly using qualitative methods. The first sub-study is a scoping review in five European countries to investigate whether and how results of research on patient preferences are considered in current procedures for coverage decisions and clinical practice guideline development. The second sub-study is a qualitative study to explore the opinions of stakeholders with regard to the possibilities for integrating evidence on patient preferences in the process of healthcare decision-making in the Netherlands. The third sub-study is the development of a decision framework for research on patient preferences. The framework will consist of: a process description regarding the place of evidence on patient preferences in the decision-making process; and a taxonomy describing different terminologies and conceptualisations of ‘preferences’ and an overview of existing methodologies for investigating preferences. The concept framework will be presented to and discussed with experts.

**Discussion:**

This study will create awareness regarding the existence and potential value of research evidence on patient preferences for healthcare policy decision-making and provides insight in the methods for investigating patient preferences and the barriers and facilitators for integration of such research in healthcare policy decisions. Results of the study will be useful for researchers, clinical practice guideline developers, healthcare policy makers, and patient representatives.

## Background

Patient preferences are increasingly considered important in healthcare policy decision-making, with many stakeholders explicitly supporting and appreciating patient involvement and consideration of patient preferences [1,2]. It is generally acknowledged that clinicians’ adherence to guidelines and the uptake of health technologies is determined by many factors, such as the clinicians’ personal beliefs and routines. Increasingly, patient-related factors such as patients’ adherence to treatment, patients’ satisfaction with treatment, and experienced (health) outcomes, all determined by patient preferences, are seen as important determinants for clinicians’ adherence to and uptake of healthcare interventions [3]. Acknowledging the importance of the patient’s perspective, a large body of research evidence on patient preferences has become available [4], and new studies on patient preferences are increasingly being funded and embedded in empirical studies, *e.g*., randomized clinical trials.

Integrating research evidence on patient preferences in healthcare policy decisions is relevant for several reasons. First, most of the outcome measures used in healthcare (economic) evaluations do not consider patients’ valuations of those outcomes. Consideration of patient preferences in healthcare policy decisions may therefore improve the uptake and real-world efficiency of healthcare technologies in its broadest sense. Second, it may serve as an important and evidence-based source of information (*e.g*., for patient representatives in healthcare decision making), therefore enhancing consumer empowerment. Third, providing doctors with results of research on collective patients’ preferences may inform individual patient preferences in the context of medical decision-making. Fourth, in general, consideration of patient views is considered as ethically the right thing to do.

Although currently there is a strong movement towards patient involvement in healthcare policy-making, systematic and explicit consideration of research evidence on patient preferences in healthcare policy decisions seems limited. Furthermore, little is known about the opinions of stakeholders towards the consideration of research evidence on patient preferences in healthcare policy decisions. Whereas some make a strong plea, or at least see possibilities, for considering research evidence on patient preferences in either coverage decisions or clinical practice guideline (CPG) development (*e.g*., [3,5-13]) the literature on this topic also suggests that there are barriers for the use of scientific data on patient preferences in healthcare policy decisions. These barriers stem from normative, psychological, methodological and practical considerations. For instance, the use of public - instead of patient - preferences for calculation of Quality Adjusted Life Years (QALY) in economic evaluations has a strong normative base [14,15]. Furthermore, the focus in health economic evaluations is on maximizing health outcomes, whereas people may attach value to aspects broader than health (*e.g*., [16]). Also, there seems to be a lack of understanding or agreement on the conceptualization and measurement of patient preferences [17]. Others doubt the quality of such research [18], doubt whether patients are capable of stating their preferences, or question the relevance of research evidence on patient preferences (*e.g*., [19]). Finally, it may be unclear how results of studies on patient preferences should be weighted against other arguments in established decision-making processes (*e.g*., [3,13]).

The aim of this paper is to describe the study protocol for an explorative study on the integration of research evidence on patient preferences in healthcare policy decisions in the Netherlands: the Patient-VIP Study (Patient Values In Policy-making Study). Results obtained in the Netherlands will function as a case study that can be used as a reference for other countries. Two types of policy decision-making will be addressed: coverage decisions (*i.e*., uptake or removal of a health technology in the benefit package) and recommendations for decision-making in CPG development. Regarding coverage decisions, the study will focus on pharmaceuticals, as in the Netherlands the decision-making process is most established for this type of healthcare technology.

For this study, the following research questions have been defined:

1. To what extent is research evidence on patient preferences considered in current procedures for coverage decisions and CPG development, and if so, how is it considered? (Study 1);

2. What are the opinions and ideas of several stakeholders with regard to development of a taxonomy for research on patient preferences, as well as the possibilities for integrating evidence on patient preferences in healthcare decision-making regarding pharmaceuticals and clinical practice guideline development in the Netherlands? (Study 2);

3. What could be the content of a decision framework for the integration of evidence on patient preferences in healthcare decision-making in the Netherlands (Study 3).

Such a framework may consist of two parts: a process description regarding the status and position of evidence on patient preferences in the decision-making processes, and an onset for a taxonomy describing the different terminologies and conceptualizations of ‘preferences,’ including an overview of existing quantitative, qualitative and mixed methods methodologies for investigating preferences. Regarding the first part of the decision framework, existing Dutch decision frameworks for coverage decisions regarding pharmaceuticals and for CPG development will be used as the starting point, with the aim to integrate the use of existing, published data on patient preferences in these existing frameworks.

The study will be predominantly based on the views, knowledge and experience of different stakeholders.

### Definitions of ‘preference

A dictionary definition of preference is: liking something better than another; tendency to choose [20]. The term ‘preference’ generally refers to the (relative) ‘desirability’ of something or someone, but is conceptualized and measured differently across disciplines. In economics, for example, the desirability of a good or service can be understood in terms of the concept of utility, referring to a measure of satisfaction gained from the consumption of a good or service, such as healthcare [14]. Utility is related to choices between goods and services, and this includes the notion of the sacrifice of one option in order to receive another. Gold and colleagues [15], p. 83 refer to preferences as numerical judgements of the desirability of a set of outcomes, and the term ‘preference’ is used interchangeably with the terms ‘utility’ or ‘value.’ In psychology, desirability is more often represented by the concept of attitude, defined as a disposition to respond favorably or unfavorably to an object, person, institution or event [21,22]. Alternatively, the term ‘preference’ may refer to an evaluative judgement in the sense of liking or disliking an object (*e.g*., [23]). In medicine, the term ‘preference’ has been defined as the desirability of a health-related outcome, process, or treatment choice [11]. As a result of different conceptualizations, varying terminology and methodology (*e.g*., [24]) is used in preference research.

In this study, the term ‘patient preference’ is broadly defined as ‘the value attached by patients to (aspects of) health and healthcare*.*’ With this work definition, we are not *a priori* excluding specific conceptualizations and/or measurement techniques, and not *a priori* confining the evaluative space to health-related outcomes only, but extend it with outcomes beyond health and process features.

### Current decision frameworks in the Netherlands

#### Coverage decisions

In healthcare systems, the aim is to make healthcare financially accessible. The challenge is to ensure that only those technologies that are safe, efficacious and effective are reimbursed [25,26]. Reimbursement decisions are made so as to ensure the greatest good for a group of patients or individuals, among others. To achieve this, the use of relevant information and knowledge is essential in the decision-making process. Informed decision-making can be enhanced by the preparation of evaluations of health interventions or health technology assessments (HTAs), which encompass aspects such as safety, efficacy, effectiveness, medical necessity, economic efficiency, social impact, and equity, as well as the quality of the evaluation itself [27]. Many countries, including the Netherlands, rely on HTA for crucial technical information. There is increasing interest in how governments actually make coverage decisions, and their outcomes (*e.g*., [28,29]).

In the Netherlands, coverage decisions are advised upon by the Dutch Health Care Insurance Board (CVZ). (In 2013, the CVZ and its tasks merged into the newly established ‘Zorginstituut Nederland.’ The ‘Zorginstituut Nederland’ has the following tasks: the execution of insurances and the health basket, improving the quality of care, and renewing care professions and education). A detailed description of the benefits and entitlements in Dutch healthcare and how these are defined can be found in ‘The health basket in the Netherlands’ [30]. Regarding pharmaceuticals, the decision-making process is largely structured and involves an assessment phase and an appraisal phase [31,32]. In the assessment phase, an analysis is made based on quantifiable criteria to decide whether, in principle, a pharmaceutical should be funded with collective resources. The assessment phase is generally guided by four so-called package principles, being ‘necessity,’ ‘effectiveness,’ ‘cost-effectiveness’ and ‘feasibility.’ The package principle ‘necessity’ refers to the question of whether a particular disease or healthcare service justifies a claim on solidarity given the cultural context, and is addressed through the concept of disease burden. The ‘effectiveness’ principle refers to the balance between positive and negative outcomes of the healthcare service, usually expressed in terms of clinical outcomes, quality of life, and aspects like user friendliness, and is assessed according to the principles of Evidence-Based Medicine (EBM) [33]. The ‘cost-effectiveness’ criterion defines whether the relation between societal costs and benefits of the healthcare service is acceptable. In accordance with the Dutch guideline for pharmaco-economic research [34], cost-effectiveness should be evaluated in an economic evaluation and expressed using a cost per QALY approach. The ‘feasibility’ of a healthcare service refers to whether it is (financially) attainable to include the service in the benefit package. This is usually addressed by means of budget impact analysis. Patient preference data could – according to the principles of EBM – be included in the effectiveness criterion (it sometimes is through measurement of quality of life in patients or user friendliness). However, the principles of EBM provide no strict methodological guidance regarding the use and format of patient preference information. The appraisal phase consists of a community review of the outcomes of the assessment phase, involving the application of non-quantifiable criteria derived from the principles of fairness and solidarity, and culminating in a definitive decision. A patient representative is a member of the CVZ appraisal committee. An important question in the context of a decision framework will be whether it is desirable that evidence of patient preferences is integrated in one of the existing package principles, or whether it should be issued separately. In the latter case, an important question is how such evidence should be weighted against the other criteria in the appraisal phase.

### Development of clinical practice guidelines

Clinical practice guidelines are statements that include recommendations intended to optimize patient care, and that are informed by a systematic review of available evidence and assessment of the benefits and harms of alternative care options [33]. Clinical practice guidelines link the practice of medicine more closely to the body of underlying evidence, shift the burden of evidence review from the individual practitioner to experts, and aim to improve the quality of care by synthesizing the available evidence and expert opinions for relevant clinical problems. As such, they are an established tool for quality improvement in clinical practice. Guidelines are systematically developed in (multidisciplinary) consensus groups, with grading, interpretation and translation of evidence into recommendations. The Grading of Recommendations Assessment, Development and Evaluation (GRADE) system for rating the quality of evidence and strength of recommendations is increasingly used [35]. The GRADE system describes four factors that affect the strength of a recommendation: the quality of evidence (*e.g*., homogeneous meta-analysis versus case studies); uncertainty about the balance between positive and negative effects (*e.g*., low versus high levels of toxicity, costs); uncertainty or variability in values and preferences (*e.g*., young patients versus old patients); uncertainty about whether the intervention represents a wise use of resources (*e.g.*, low versus high costs, favorable versus unfavorable budget impact analysis). A strong recommendation means that most informed patients would choose the recommended management, and that clinicians can structure their interactions with patients accordingly. A weak or so-called conditional recommendation means that patients’ choices will vary according to their values and preferences, and clinicians must ensure that patients’ care is in line with their values and preferences.

In the Netherlands, the Dutch Council for Quality of Health Care had the task of coordinating the development of CPGs. (In January 2013, the Regieraad was merged into the newly established ‘Zorginstituut Nederland’ as the ‘Quality Institute’). CPGs are developed by different guideline developers, such as the Dutch Institute for Healthcare Improvement (CBO), professional societies, the Dutch Association of Medical Specialists, the oncology collaborative groups, and Trimbos Institute for Research on Mental Health. The process of CPG development follows the principles of EBM and the criteria of the Appraisal of Guidelines for Research and Evaluation (AGREE) Instrument [36] as guidance. An evidence-based guideline is developed according to an established process and based on three sources of knowledge: best available knowledge and evidence; expertise, experiences and preferences of healthcare practitioners; and experiential knowledge, experiences and preferences of patients. The AGREE instrument makes an explicit reference to research evidence on patient experiences as a potential source of information. However, as noted, the principles of EBM provide no strict methodological guidance regarding the use and format of patient preference information.

## Methods

### Study design

This study consists of three sub-studies.

### Study 1: scoping review

The aim of the scoping review is to investigate whether and how results of research on patient preferences are considered in current procedures for coverage decisions and CPG development. We will search several bibliographic databases and websites for relevant (gray) literature, as well as reports and documents for the Netherlands, Germany, England and Wales, Scotland and France with the aim to get insight in current procedures for coverage decisions and guideline development, as well as their reference to research on patients’ preferences. These countries are selected because of relevant similarities and differences regarding the financing and regulation of their healthcare system. Furthermore, we will study the contents of selected coverage decisions and CPG regarding some major clinical topics on their reference to research on patient preferences. The scoping review will reveal whether the integration of evidence on patient preferences in healthcare policy decisions differs between countries. Furthermore, if such evidence is considered in either one of the countries, it may inform the development of a decision framework the Netherlands.

### Study 2: qualitative study

The aim of the qualitative study is to explore opinions and ideas of stakeholders with regard to the term ‘preference,’ as well as the relevance of research evidence on patient preferences for healthcare policy decisions. Potential strategies to resolve the barriers and enhance facilitators for the integration of patient preference data in healthcare policy decisions will also be issued during the interviews. First, in-depth semi-structured interviews will be held with key-informants from several Dutch stakeholders such as researchers, patient representatives, guideline developers, and healthcare policymakers. We aim for a heterogeneous sample of participants with different perspectives and ideas on how to incorporate evidence on patient preferences in healthcare decision-making regarding pharmaceuticals. Although we aim for interviews with stakeholders that can reflect on both coverage decision making and guideline development, we will also turn to specialists on one of these two fields. The face-to-face or phone interviews will be open and will be characterized by a personal approach, meaning that the interviewer has some knowledge of the background and work of the person to be interviewed, ensures that the objective and procedure of the study are clear, and stimulates the participant to express his or her opinion by explaining that there are no good or wrong answers and that each opinion or idea will be included in the analysis. After the introduction, the interviewers will follow the interview scheme, based on a list of themes and examples to ensure that all relevant items are brought up during the discussion. Interviews will be audio-recorded and transcribed verbatim. An independent senior researcher will carry out the interviews (CU). Member-checking will be done by sending interview participants a summary of the main findings, extracted from their single interviews.

The interviews will be analyzed by directive content analysis [37]. The data will be divided into simpler text units for coding that will be entered into a database (Nvivo). Units of text referring to similar codes will be grouped and categorized systematically by one central coder, who is coding all the interviews (CU). For the most informative interview - in the opinion of the interviewer - of each subset of interviews, a full open coding of the transcript will be independently executed by the central coder (*i.e*., interviewer) and three members of the project group (CD, MJ, TvdW). Differences in coding will be resolved by consensus discussion, face-to-face or by phone. The central coder will then analyze the other interviews.

### Study 3: development of a draft decision framework

In the third study, we will develop a draft framework for decision-making, based on sub-studies 1 and 2. The framework will consist of a process description regarding the place of evidence on patient preferences in the decision-making process, and a taxonomy describing different terminologies and conceptualizations of ‘preferences,’ including an overview of existing methodologies for investigating preferences. As we expect that the qualitative interviews will not provide detailed input to enable development of a final taxonomy, we will perform a (non-systematic) review in which the most prominent, well-known theoretical or conceptual models for preferences and decision-making will be included. These will include, among others, utility theory [38], health behavior models [39], the behavioral economic model for decision-making [40], and satisfaction models [41,42]. Based on this review, a systematic categorization of related terminologies, conceptualizations and definitions will be given. Furthermore, we will build on an existing review [24] to provide an overview of methodologies for measurement of preferences. With the draft taxonomy, we aim to connect the different terminologies and conceptualizations of preferences to existing measurement techniques. Furthermore, based on sub-studies 1 and 2, we will develop the process description part of the decision framework, listing recommendations regarding consideration of results of research on patient preferences, either in coverage decisions or CPG development.

The concept decision framework will be sent to experts. We will sample well-known Dutch opinion leaders and experts again from different stakeholders groups. At least one expert meeting (about 10 participants), which may take the form of an invitational conference, will be organized to present the draft decision framework, to discuss the framework, to identify the most critical parts and issues, and to define necessary steps towards further development, validation and implementation of the framework.

### Study status

The study protocol was presented to the medical ethics committee of the Maastricht University Medical Centre. The committee concluded that formal approval was not required for the three sub-studies.

The first two sub-studies of the study are in progress. The scoping review is in its final stage. The last interviews are currently being performed.

## Discussion

We presented the research protocol of a project with three sub-studies focussing on the integration of results of research on patient preferences in consensus and decision-making processes of coverage decisions and clinical practice guideline development. This study will create awareness regarding the existence and potential value of research evidence on patient preferences for healthcare policy decision-making, and provides insight into the methods for investigating patient preferences and the barriers and facilitators for integration of such research evidence in healthcare policy decisions. An important prerequisite for this integration of research evidence on patient preferences in healthcare policy decisions is an overview (a taxonomy) of what may constitute evidence in this context. Ultimately, our aim is to develop a framework that will assist decision-makers in considering this type of information in coverage decisions and GPG development. We feel that this is the right time to take up the challenge and to see how established decision-making processes regarding coverage decisions and guideline development can be adapted in such a way that research on patient preferences gets a fair weight in policy decisions. In the end, systematic and explicit use of results of research evidence on patients’ preferences is the ultimate and only justification for investing in such research either by governments through allocation of research budgets or by patients through their time and effort.

The strength of this study is that we will first describe a status quo measurement that will be the starting point for the qualitative interviews and development of the decision framework. Furthermore, this study focuses both on coverage decisions and on CPG development; increasingly, it is recognized that both ‘worlds’ are to be connected and that decision-making on these topics should be based on the same arguments. This view is, for example, reflected by the opinion that cost-effectiveness arguments should systematically be considered in CPG development [43]. Therefore, several experts within the fields of health technology assessment/health economics, healthcare policy-making, evidence-based medicine, and guideline development will be consulted. A limitation is that this study, except for the scoping review, is performed within the Dutch context and results may therefore not directly be transferable to other countries.

Results of this study will be useful for healthcare policy decision makers (*e.g*., members of assessment and appraisal committees of coverage decision-making, or advising organizations, ministries of health, guideline developers, and patient platforms). The target population also consists of researchers. First, the study may stimulate collection of data on patient preferences in routine healthcare (economic) evaluations when relevant. Second, the decision framework may put specific demands on future research on patient preferences. Patient organizations are a specific target group. The decision framework may also stimulate the professional staff of a patient organization to collect or retrieve the evidence on patient preferences in line with the taxonomy, so they are always well-prepared for the task of representing their patient group. During the project, stakeholders are actively involved through the use of semi-structured interviews and expert meetings, the latter to reach consensus on the contents of the decision framework.

This study is not about active participation of patients in the process of coverage decisions and CPG development, but on reaching consensus and improving these decision processes by systematically and explicitly considering existing research evidence on patient preferences. Currently, much attention is devoted to innovative methods to engage patient and public representatives in healthcare decisions, which is seen as important by many stakeholder groups [1,2]. However, patient participation in coverage decisions or CPG development is not a substitute for systematically considering research evidence on patient preferences in healthcare decisions (or vice versa). On the contrary, providing patient representatives (and other decision-makers) with evidence on patient preferences is expected to result in better decisions.

Given the complexity of the empirical research on patient preferences, stemming from various disciplines using completely different frameworks and methodologies, it is expected that this study will result in a first draft and not in a definite, ready to use decision framework. Ultimately, we seek to deliver some bricks with the aim of building a practical ‘decision framework’ for the integration of evidence in patient preferences in healthcare policy decisions. The taxonomy part of the decision framework will need improvement and validation in an international context. We also realize that creating a consistent language for preference-related research and providing a systematic categorization for the types of preference-related research will not be sufficient. In order to judge research on patient preferences, we need quality assessment criteria for appraising such studies and an additional step of evidence synthesis and grading. Although quality criteria have already been developed, *e.g*., for conjoint analysis studies (*e.g*., [44,45]), they may be too unspecific with regards to other preference elicitation methods. Furthermore, even if we are able to provide all the necessary ingredients for the decision framework, it will not provide explicit guidance on how to weigh evidence on patient preferences against other arguments in the decision-making process. Despite these limitations, the first draft of the decision framework may serve as an important starting point for future integration of evidence on patient preferences in healthcare policy decision making.

## Abbreviations

CBO: Dutch Institute for Healthcare Improvement; CPG: Clinical practice guidelines; CVZ: Dutch healthcare insurance board; EBM: Evidence-based medicine; HTA: Health technology assessment; NPCF: Dutch Federation of Patients and Consumer Organisations; QALY: Quality adjusted life years.

## Competing interests

The authors declare to have no competing interest regarding this study.

## Authors’ contributions

CD is project leader. MJ and TvW are co project leaders. CD, MJ and TvW designed the study and received a grant. CU is the main researcher on the project and will perform the sub-studies. CD wrote the manuscript. MJ, CU and TvW contributed to the writing of the manuscript. BB, CS, DD, HL and TB are advisors for the project. AS is member of the project committee. All authors reviewed and provided feedback for this manuscript. The final version was approved by all authors. All authors read and approved the final manuscript.
